# Kinetic parameters of alpha-synuclein seed amplification assay correlate with cognitive impairment in patients with Lewy body disorders

**DOI:** 10.1186/s40478-023-01653-3

**Published:** 2023-10-09

**Authors:** Stefan Bräuer, Marcello Rossi, Johann Sajapin, Thomas Henle, Thomas Gasser, Piero Parchi, Kathrin Brockmann, Björn H. Falkenburger

**Affiliations:** 1 Department of Neurology, University Hospital Carl Gustav Carus, Technische Universität Dresden, Dresden, Germany; 2 German Center for Neurodegenerative Diseases (DZNE), Dresden, Germany; 3 IRCCS Istituto delle Scienze Neurologiche di Bologna (ISNB), Bologna, Italy; 4 Department of Food Chemistry, TU Dresden, Dresden, Germany; 5 Department of Neurodegenerative Diseases, Hertie Institute for Clinical Brain Research, Eberhard Karls University Tübingen, Tübingen, Germany; 6 German Center for Neurodegenerative Diseases (DZNE), Tübingen, Germany; 7 Department of Biomedical and Neuromotor Sciences, University of Bologna, Bologna, Italy

**Keywords:** Alpha-synuclein, Dementia with Lewy bodies, Parkinson’s disease, RT-QuIC, Seed amplification assay

## Abstract

The alpha-synuclein (aSyn) seed amplification assay (SAA) can identify aSyn aggregates as indicator for Lewy body pathology in biomaterials of living patients and help in diagnosing Parkinson´s disease and dementia syndromes. Our objective was to confirm that qualitative results of aSyn SAA are reproducible across laboratories and to determine whether quantitative findings correlate with patient clinical characteristics. Therefore cerebrospinal fluid samples were re-analysed by aSyn SAA in a second laboratory with four technical replicates for each sample. Kinetic parameters derived from each aggregation curve were summarized and correlated with patient characteristics. We found that qualitative findings were identical between the two laboratories for 54 of 55 patient samples. The number of positive replicates for each sample also showed good agreement between laboratories. Moreover, specific kinetic parameters of the SAA showed a strong correlation with clinical parameters, notably with cognitive performance evaluated by the Montreal Cognitive Assessment. We concluded that SAA findings are highly reproducible across laboratories following the same protocol. SAA reports not only the presence of Lewy pathology but is also associated with clinical characteristics. Thus, aSyn SAA can potentially be used for patient stratification and determining the target engagement of aSyn targeting treatments.

**Supplementary Information:**

The online version contains supplementary material available at 10.1186/s40478-023-01653-3.

## Introduction

The deposition and propagation of pathologically aggregated alpha-synuclein (aSyn) is a central component in the pathogenesis and progression of Parkinson’s disease (PD) and other neurodegenerative disorders that are collectively called *synucleinopathies*. Diagnosis of PD and other synucleinopathies is currently based mainly on clinical criteria [1], so the diagnostic accuracy in early PD is not optimal [2]. Moreover, the course of PD is quite variable [3], so biomarkers to support early diagnosis and prognostication are direly needed.

Seed amplification assays (SAAs) have been established to detect pathological conformations of prion protein [4] and modified to detect pathological conformations of aSyn in brain extracts, biopsies (skin, olfactory mucosa, submandibular glands) [5–7], cerebrospinal fluid (CSF) [8–10], blood [11, 12] and saliva [13]. SAAs separate patients with PD and Dementia with Lewy bodies (DLB) from healthy controls with high sensitivity and specificity [14–16] and also allow diagnosis of synucleinopathies in the prodromal stage, such as pure autonomic failure or idiopathic REM sleep behavior disorder [14, 17, 18].

There is considerable diversity among SAA protocols, and many different assay variants have been described [19]. Yet, standardization becomes important as techniques move from scientific exploration into clinical routine. We implemented an established protocol in a new setting to demonstrate that reproducible results can be obtained between laboratories and compared the qualitative findings. Moreover, we explored the possibility that quantitative parameters of the aggregation assay reflect clinical characteristics.

## Materials and methods

### Study population

To analyze the reproducibility of qualitative findings between laboratories, we used a subset of 55 patient samples described previously [16] (45 with a clinical diagnosis of DLB and 10 PD), which were collected between 2006 and 2020 at the University of Tübingen, and for which aSyn seeding activity was measured at the Istituto delle Scienze Neurologiche di Bologna (ISNB). The initial aim for a successful reproduction was an agreement of at least 95%. We estimated that 55 samples would be sufficient (3/4 positive at ISNB, 1/4 negative at ISNB). We did not perform a sample size calculation beforehand but analyzed the results using kappa statistics. The Tübingen team selected samples to represent a spectrum of Lewy body diseases. After an initial correlation of assay parameters with phenotypical characteristics was observed, we conducted a sample size calculation in which we expected a moderate correlation (rho = 0.4, alpha = 0.05, power = 0.9) and collected 20 additional samples at Technische Universität Dresden (TUD) between 2020 and 2022. This study was approved by the institutional review board of TUD (BO-EK-444092021), and written informed consent was obtained from all participants before inclusion in the study. Overall, samples from 75 patients with a diagnosis of Lewy body disease (PD or DLB) were included in the analysis. Patient characteristics are summarized in Table 1; data for individual patients and SAA results are listed in Additional file 1: Table S1.

**Table 1:** Patient characteristics

	All	PD	DLB
Number	75	28	47
Sex (m/f)	56/19	20/8	36/11
Age	69.44 ± 8.94	64.62 ± 10.63	72.37 ± 6.22
Age at onset	64.93 ± 9.6	59.57 ± 11.06	68.27 ± 6.81
Disease duration	3.64 ± 2.4	3.89 ± 2.75	3.49 ± 2.18
MoCA	18.36 ± 7.85	25.21 ± 4.95	13.32 ± 5.35
UPDRS-III	24.88 ± 12.73	21.19 ± 10.54	31.13 ± 13.98

*PD* Parkinson’s disease; *DLB* Dementia with Lewy bodies; *f* Female; *m* Male; *MoCA* Montreal cognitive assessment; *UPDRS-III* Unified Parkinson’s disease rating scale part III

Data depicted as mean ± standard deviation

A movement disorders specialist examined all participants. For PD, the diagnosis was made according to the UK Brain Bank Society Criteria [20]; DLB was defined according to the DLB consortium revised consensus criteria [21]. The severity of motor symptoms was taken from patient charts and assessed in motor ON by the Unified Parkinson’s Disease Rating Scale part III (UPDRS-III), using the original version between 2006 and 2008 and the MDS-UPDRS from 2009 on [22]. Cognitive performance was assessed in routine care using the Mini Mental Status Examination (MMSE) [23] or the Montreal Cognitive Assessment (MoCA) [24]. Scores from the MMSE were converted to MoCA equivalents as previously described [25]. Cognitive impairment was defined as a MoCA score below 24 [26].

Lumbar puncture was performed using standard protocols. CSF samples were centrifuged and frozen at − 80 °C within 90 min after collection. For blinding, samples were shipped between laboratories with identification by numeric patient IDs and unblinded only after the measurements. For samples collected at the same site (TUD), those were labeled by numeric IDs by an investigator not involved in the analysis and only unblinded after the measurements.

### Human alpha-synuclein protein purification

One investigator from TUD (SB) was trained at ISNB. The original subset of n = 55 CSF samples has been already measured at ISNB [16]. In both laboratories (ISNB, TUD), synthesis, purification of recombinant wild-type (WT) human aSyn and the SAA assay were conducted as established at ISNB [14] with only minor modifications at TUD. We produced two different types of aSyn monomer batches with different kinetics in the SAA, which we termed “slow-kinetic”-batches and “fast-kinetic”-batches. Briefly, BL21 (DE3) E. coli bacteria (Thermo Fischer Scientific) were transformed with the vector plasmid containing the WT human aSyn. For the “fast-kinetic” batch, the aSyn ORF was cloned from pT7-7 aSyn WT (gift from Hilal Lashuel, Addgene plasmid #36,046) into pET-28a(+) (Merck) using the restriction sites HindIII and NdeI. Expression was induced via the Overnight Express Autoinduction System (Merck), and cells were harvested after 18 h. For the “slow-kinetic” batch, the N-terminal-His aSyn construct was cloned from pET-28a(+) WT aSyn into pT7-7 using HindIII and XbaI. Induction was performed by adding 1 mM Isopropyl-β-D-thiogalactopyranoside (Carl Roth) after the culture reached an OD600 of 0.8. Cells were harvested by centrifugation 6 h later. The protocol used after harvesting cells was identical for both batches. The cell pellet was lysed via osmotic shock [400 g/l sucrose (Carl Roth), 30 mM TRIS (Carl-Roth) pH 7.2, 2 mM EDTA (Thermo Fischer Scientific)]. The solution was centrifuged and the pellet resolved in water. After centrifugation, the supernatant’s pH was reduced to 3.5. The solution was centrifuged again and the supernatant’s pH increased to 7.5. Next, we performed immobilized metal ion affinity chromatography using an NGC chromatography system (BioRad) and HisTrap FF-column (Cytivia). The selected fraction was loaded on a HiTrap Q-HP anion exchange-column (Cytivia), the selected fractions pooled and subsequently dialyzed against water using a 3.5 kDa MWCO dialysis membrane (Thermo Fisher Scientific) overnight at 4 °C. The protein concentration was measured using a spectrometer (NanoDrop, Thermo Fischer Scientific). The samples were lyophilized and stored at − 80 °C until further use. Each batch was validated – with comparable results displayed in Additional file 2: Fig. S1. The “slow-kinetic” batches were used for all measurements unless noted otherwise.

### Alpha-synuclein seed amplification assay

The aSyn assay was performed as previously described [14]. In brief, measurements were performed using a black 96-well plate (Thermo Fischer Scientific). Each well contained six 0.8 mm silica beads (OPS Diagnostics). Per well, 15 µl of CSF was added to 85 µl of reaction buffer, which contained 40 mM phosphate buffer (Carl Roth) pH 8.0, 0.0015% sodium dodecyl sulfate (SDS)(Carl Roth), 10 µM Thioflavin T (Carl Roth), 0.1 mg/ml recombinant aSyn, 170 mM NaCl (Carl Roth). The plate was incubated in a BMG FLUOstar Omega plate reader at 42 °C with cycles of 1 min double orbital shaking (400 rpm) and 1 min rest. The fluorescence measurements were performed every 45 min.

Each sample was measured as four technical replicates on the same plate. Each plate included at least one negative control and two positive controls, each with four replicates. Relative fluorescence units (RFU) for every time point were expressed as a percentage of the maximum intensity reached on that plate. A replicate was considered positive if fluorescence crossed a threshold within a given time window. The window was set to 30 h at ISNB. Using the “slow-kinetic” aSyn monomer, it was set to 40 h at TUD. The fluorescence threshold was defined as the average intensity of previously measured negative controls during the first 10 h of recording, plus 40 standard deviations. A sample was considered positive if at least two replicates were positive and was considered negative if no replicate was positive. A sample was run again if one replicate was positive; in this way, two samples were measured twice, and four samples were measured three times. In positive samples, the following kinetic parameters were determined for each positive replicate: Area under the curve (AUC), the peak of the fluorescence response (Imax), the lag phase (LAG), i.e., the time to reach the threshold. For each sample, these parameters were summarized by calculating the mean of all positive replicates. The parameter TT2 (“Time to 2 positive replicates”) was defined as the second shortest LAG, TT1 (“Time to 1 positive replicate”) was defined as the shortest LAG.

### Data analysis and statistics

Data was analyzed using GraphPad Prism 9.0.0, RStudio version 4.2.3 and the following packages: corrplot, DescTools, dplyr, factoextra, FactoMineR, ggplot2, hablar, pwr, readxl, stringr, tidyr. Spearman´s rho was used for correlation since data was non-normally distributed; after evaluation of the clinical scores used (MoCA, UPDRS-III) and scatterplots, we presumed non-linearity and therefore fitted the data by second-degree polynomial regression (Fig. 2, Additional file 2: Fig. S2 and S3), using ggplot2. Shapiro wilks test was used to test for normal distribution, Mann–Whitney U test was used to compare non-normally distributed data and t-test to compare data with normal distribution. The agreement of qualitative SAA results was quantified with Cohen´s kappa. The principal component analysis (PCA) used the stats package; visualization was performed by *corrplot*. A linear model (*lm*, stats package) was used to determine whether TT2 is better predicted by MoCA or UPDRS-III. To determine the optimal cut-off to predict cognitive impairment (defined as MoCA < 24) by the kinetic feature TT2, we performed a ROC analysis using Microsoft Excel 2016. R-codes are listed in the Additional file 3.

## Results

### Reproducibility of assay results between laboratories

To test the reproducibility of SAA results across laboratories, we newly established at TUD the protocol of ISNB, and re-tested a diverse set of 55 patient samples previously measured at ISNB [16] in a blinded fashion. The qualitative result was reproduced in 54 out of 55 samples (98%, 12 negatives, and 43 positives, see Additional file 2: Table S2), suggesting that the qualitative results of the SAA are highly reproducible when trained investigators use the same protocol (Cohen´s kappa = 0.948, 95% CI = 0.848–1.000).

**Fig. 1:**
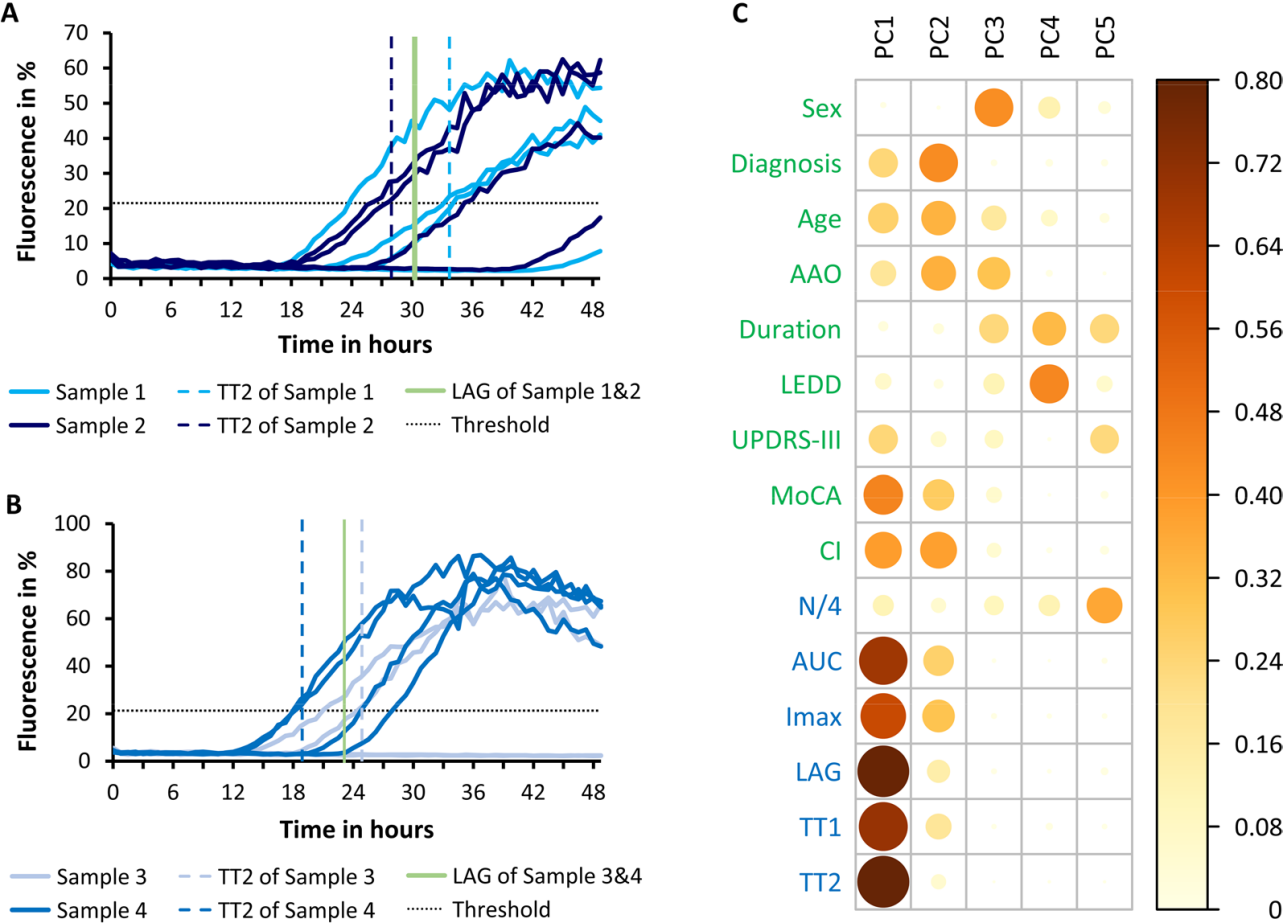
Kinetic features of SAA measurements. **A** SAA aggregation curves of 2 samples with 4 replicates each. Samples show a similar average lag phase (30 h and 31 h (light green line)) and the same number of positive replicates (3 of 4) but quite a different second fastest lag phase (TT2, 34 h and 28 h (dashed lines)). **B** SAA aggregation curves of 2 samples with 4 replicates each. Samples show a similar average lag phase (23 h and 23 h (light green line)) even though the numbers of positive replicates are quite different (4 of 4 and 2 of 4). TT2 better reflects possible differences (19 h and 25 h (dashed lines)). **C** Principal component analysis of clinical features (green) and kinetic features as obtained at TUD (blue). For each component (PC1-PC5), the loadings for each feature (cos^2^) are expressed color-coded as indicated by the legend. Features contributing strongly to a component are marked by large and dark circles. Abbreviations: Diagnosis, PD or DLB; AAO, age at onset; duration, disease duration; LEDD, levodopa daily dose; UPDRS-III, Unified Parkinson’s disease rating scale part III (motor); MoCA, Montreal cognitive assessment; CI, cognitive impairment (presence of); TUD, kinetic parameters measured at TUD; N/4, number of positive replicates (out of four); AUC, area under the curve; Imax, maximum fluorescence intensity; LAG, mean lag phase; TT1, fastest lag phase; TT2, second fastest lag phase

We measured each sample as four technical replicates (Fig. 1A, B) and considered positive those reaching the threshold (dotted lines in Fig. 1A, B) within a given time frame. We analyzed whether this parameter also constitutes a comparable property of a patient sample between laboratories. An identical result was obtained in 34 of 55 samples (62%, see Additional file 2: Table S3 for details).

### Kinetic parameters correlate with cognitive performance

The aggregation of each replicate measurement can be described by a number of kinetic features, including AUC, Imax and LAG. To obtain an overview, we compared the association between the patient’s clinical characteristics (green in Fig. 1C) with kinetic features obtained at TUD (blue in Fig. 1C) using principal component analysis. A subset of clinical characteristics loaded mainly on the first component (PC1), including cognitive performance as reported by MoCA, and the presence of cognitive impairment (CI), but not age, age at onset (AAO), disease duration, diagnosis (PD vs. DLB), daily levodopa dose (LEDD) or sex. Several kinetic features also loaded on PC1, particularly the average lag phase (LAG) and the second shortest lag phase (TT2). The close association of kinetic features suggests that a single parameter could represent them. The closest association with clinical features was observed for TT2, a median-like feature that varies over a wider range of values than the fastest lag phase (TT1) and the average lag phase (LAG). Hence, TT2 can differ between samples with the same LAG (Fig. 1A) and is influenced less by the number of positive replicates (Fig. 1B).

**Fig. 2:**
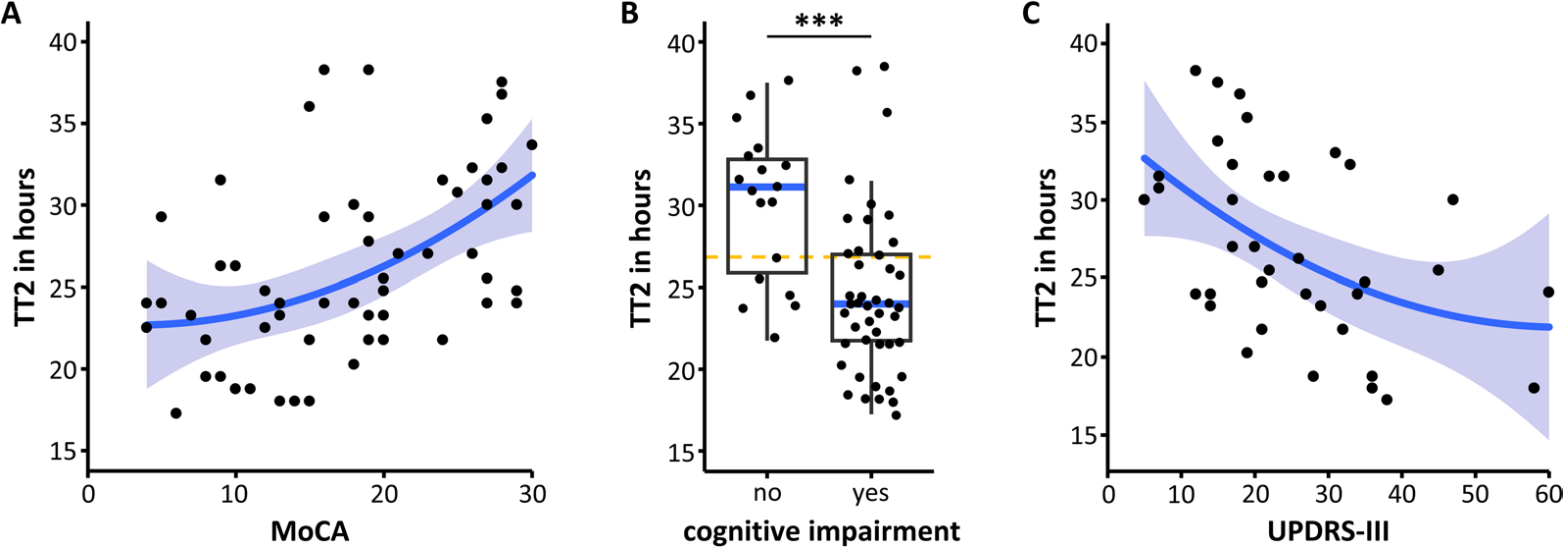
Correlation of the second fastest lag phase with clinical features. **A** Correlation of the second fastest lag phase (TT2) with cognitive performance as quantified by the Montreal cognitive assessment (MoCA). **B** TT2 for patients with and without cognitive impairment, defined as MoCA < 24. Comparison by Mann–Whitney U test, r = 0.482, *p* < 0.001. The orange dashed line indicates the 27h threshold used for the calculation of sensitivity and specificity described in the text. **C** Correlation of TT2 with motor symptoms reflected by the Unified Parkinson’s disease rating scale part III (UPDRS-III). In A-C, each marker represents one patient sample. In A and C, nonlinear regression and 95% confidential interval are indicated by blue line and blue-grey area. In B, box indicates 25 and 75 percentiles, whiskers indicate the range, blue line indicates median

We then explored the association between kinetic features and clinical characteristics in more detail, finding a strong positive correlation between TT2 and cognitive performance measured by MoCA (Spearman´s rho = 0.519, 95% CI = 0.297–0.688, *p* < 0.0001; Fig. 2A). Accordingly, we observed a significant difference between TT2 of patients with and without cognitive impairment (Mann–Whitney U test, r = 0.483, *p* < 0.001; Fig. 2B). In the ROC analysis for detecting cognitive impairment with a TT2-threshold, we identified 27 h as the optimal border to differentiate between cognitively impaired and unimpaired patients, using TT2 as the SAA kinetic feature. With this threshold, we obtained a sensitivity of 73% and a specificity of 72% for detecting cognitive impairment (ROC-AUC 0.82). Analyzing PD and DLB patients separately, we still found a significant correlation between TT2 and MoCA, which was strong in PD patients (Spearman´s rho = 0.550, 95% CI = 0.153–0.793, *p* = 0.008) and moderate in DLB (Spearman´s rho = 0.335, 95% CI = 0.003–0.601, *p* = 0.043). We also observed a moderate correlation of MoCA with LAG, both for the entire cohort and for the PD and DLB subpopulations (Spearman´s rho = 0.412, 95% CI = 0.167–0.609, *p* < 0.01; PD: Spearman´s rho = 0.500, 95% CI = 0.087–0.767, *p* = 0.018; DLB: Spearman´s rho = 0.349, 95% CI = 0.018–0.611, *p* = 0.034; Additional file 2: Figure S2A).

For motor symptoms reported by the UPDRS-III, we observed a moderate negative correlation with TT2, as a shorter TT2 was associated with higher UPDRS-III (Spearman´s rho = − 0.469, 95% CI = − 0.694 to − 0.161, *p* < 0.01; Fig. 2C). UPDRS-III also correlated with MoCA (Spearman´s rho = − 0.597, 95% CI = − 0.766 to − 0.349, *p* < 0.001; Additional file 2: Fig. S3), so we were wondering whether the SAA kinetic features better represent motor symptoms (UPDRS-III) or cognitive impairment (MoCA). We used linear models to address this question. MoCA and UPDRS-III were significant factors when included individually in a linear model to predict TT2 (ANOVA, F = 13.25, *p* = 0.001 and F = 10.04, *p* = 0.003). Yet, MoCA was a significant factor when added after UPDRS-III (ANOVA, F = 5.76, *p* = 0.023), whereas UPDRS part III was not (ANOVA, F = 2.56, *p* = 0.120). This suggests that TT2 is more closely associated with MoCA than with UPDRS-III, and the correlation between motor symptoms and cognitive impairment might in part explain the correlation between SAA kinetic features and motor symptoms.

### Slow assay kinetics support resolution of clinical characteristics

**Fig. 3:**
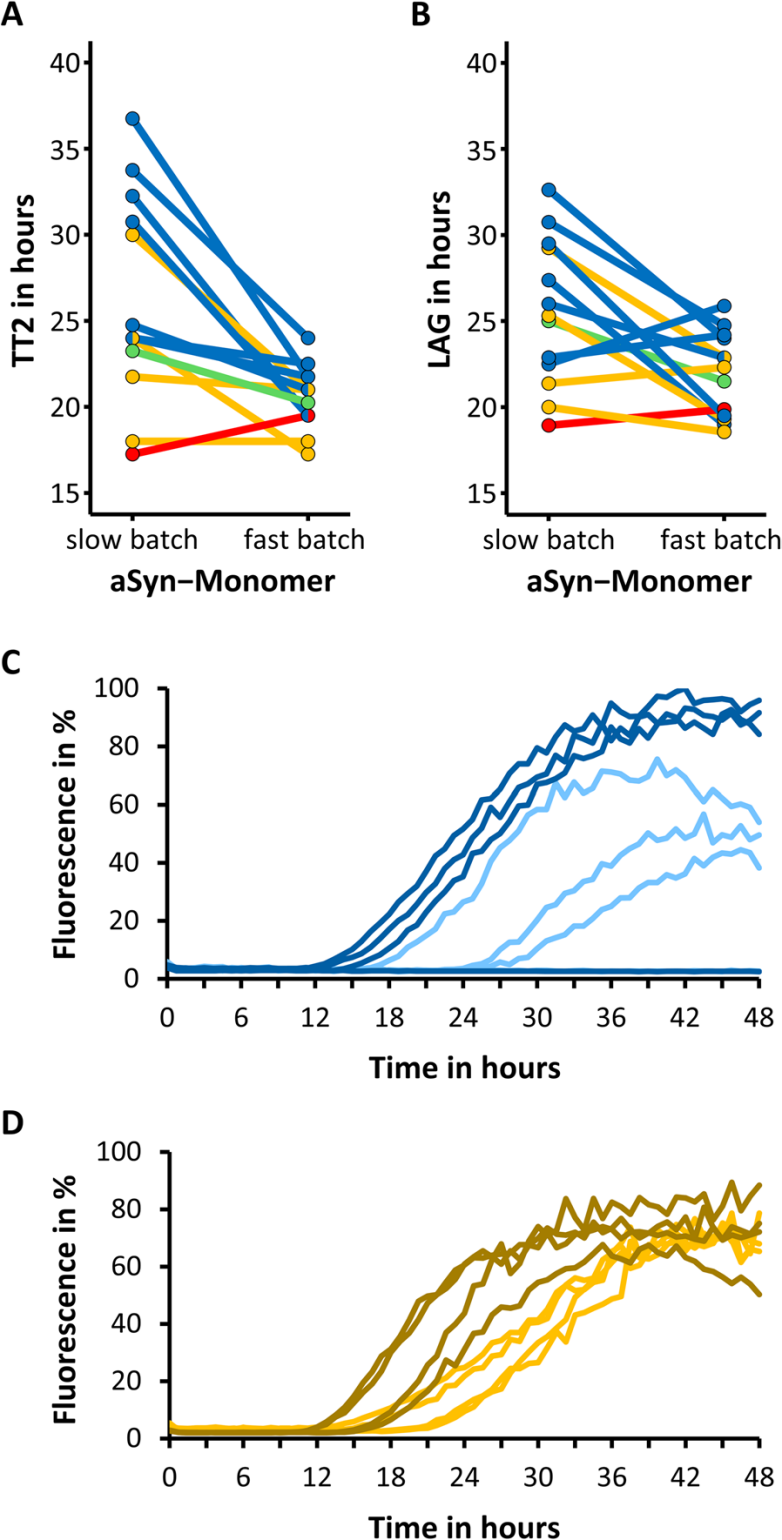
Impact of alpha-synuclein monomer on SAA kinetic features. **A** Alpha-synuclein (aSyn) monomer-dependent change of the second fastest Lag phase (TT2). **B** aSyn monomer-dependent change of average Lag phase (LAG). In A and B, each line represents one CSF-sample (13 in total) which was measured with both, the “slow-kinetic”- and “fast-kinetic”-aSyn monomer. Patients’ MoCA-scores are coded by color: MoCA ≥ 25, blue; MoCA 24–20, green; MoCA 19–15, orange; MoCA < 15, red. **C** SAA aggregation curves of one sample (MoCA ≥ 25) with 4 replicates, measured with “slow-kinetic”- (light blue) and “fast-kinetic”-aSyn monomer (dark blue). **D** SAA aggregation curves of one sample (MoCA 19–15) with 4 replicates, measured with “slow-kinetic”- (orange) and “fast-kinetic”-aSyn monomer (dark orange)

Finally, we explored possible explanations why the association of clinical characteristics with kinetic features was not observed as strongly in a previous study on the cohort including the patients we tested. We noticed that kinetics obtained here were somewhat slower than previously (average LAG of all 43 positive samples at ISNB 19.78 ± 2.95 h (SD) vs. average LAG of all 42 positive samples at TUD 25.73 ± 4.36 h (SD)). We therefore hypothesized that a fast and efficient seeding assay, as observed in the previous work could make it harder to resolve small differences between individual patients. Indeed, the SAA has previously been optimized for the qualitative differentiation between patient samples with and without Lewy pathology. To test this hypothesis, we measured a subset of samples with two different monomer preparations that differ in the TT2 and average LAG (Fig. 3). These monomers were purified using different induction-methods of protein expression as described in the methods section. More homogenous results were obtained with the “fast-kinetic” aSyn monomer and more variation was observed using the “slow-kinetic” aSyn monomer as substrate in the SAA (range of 20 h vs 7 h between minimum and maximum TT2). This had a relevant impact on the representation of patients’ cognitive performance (MoCA) by the SAA kinetic parameters, such as TT2.

## Discussion

In this study, we demonstrated that the qualitative detection of Lewy pathology by the aSyn SAA is highly reproducible across laboratories. Quantitative features of the assay correlate with phenotypical patient characteristics, particularly cognitive performance.

The reproducibility of qualitative SAA results has been investigated between methods [27] run in the same laboratory and between different assays that are commercially available [15]. Our findings confirm the findings in an academic setting using the same assay protocol. To ensure that the high sensitivity and specificity described by leading SAA laboratories [9, 10, 14, 15] can be extended to the clinical setting, interlaboratory ring tests and further quality assurance measurements need to be established, as implemented for sporadic Creutzfeldt-Jakob disease [28, 29].

The SAA comprises several steps, i.e., monomer production, the aggregation measurement itself, and the experimental design, i.e., the number of technical replicates and the criteria defining a positive result. Our findings demonstrate that minor changes in monomer production can alter assay kinetics (Fig. 3). For qualitative findings, such differences can be compensated by adjusting the experimental design (in this case, the time window allowed to cross the fluorescence threshold), highlighting the importance of testing the entire chain of events in ring trials.

In the present work, we observed systematic differences in aggregation kinetics with different protocols for aSyn monomer preparation (Fig. 3). Monomer preparations that produce fast aggregation curves showed a shorter and more homogenous LAG, than monomer preparations that produce slower aggregation curves (Fig. 3A, B). Fast aggregation curves appeared compressed into a “floor” with respect to LAG, resulting in less variable LAG (Fig. 3C). While fast and uniform kinetics are optimal for the qualitative detection of Lewy pathology with high sensitivity and specificity, allowing slower and more variable aggregation kinetics in a research setting could better resolve differences in seeding activity between individual patients.

Among the clinical characteristics investigated here, cognitive performance correlated best with SAA features, such as TT2 (Fig. 1C). This is somewhat surprising given that motor symptoms are often more prominent in patients with Lewy pathology. UPDRS-III values varied between 5 and 60, ruling out floor or ceiling effects as an explanation. Indeed, motor symptoms reflect degeneration of dopaminergic neurons in the substantia nigra, which is not closely correlated with the extent of Lewy pathology in the brain [30]. Moreover, a large fraction of dopaminergic neurons is already lost at the time of diagnosis [31], and UPDRS-III scores show limited precision in capturing disease progression in early PD [32]. In contrast, cognitive impairment emerges as Lewy pathology spreads to limbic and neocortical areas, so cognitive performance reflects the extent of Lewy pathology in a large part of the brain [33, 34]. This might explain why cognitive impairment, as reported by MoCA, better correlates with SAA features. Yet, SAA features did not explain all of the cognitive performance (e.g., outliers in Fig. 2A and PC2 in Fig. 1C) and additional pathologies like amyloid-beta and metabolic factors contribute to cognitive decline [35–37].

In this study, we have mainly combined samples from patients with PD and DLB because they can be considered parts of a spectrum of Lewy body diseases. The correlation of kinetic features with MoCA was observed also when analyzing samples from patients with PD and samples from patients with DLB separately, confirming the validity of the measurements for each individual disease. Interestingly, the correlation between TT2 and MoCA was strong in PD samples and moderate in DLB samples. Indeed, the nonlinear regression of TT2 vs. MoCA was steeper for MoCA scores > 20 than for MoCA scores < 20 (Fig. 2A) and the average MoCA of patients with DLB was lower than for patients with PD (Table 1). Nonlinear properties of the MoCA score have been reported [38, 39], but longitudinal studies will be required to resolve the origin of this observation. Thus, our cross-sectional dataset suggests that SAA could be suited to report quantitative differences in cognitive performance during the early phases of cognitive decline (MoCA 20-30).

In this study, we investigated quantitative differences in aggregation kinetics between samples obtained from different patients with Lewy body disorders. On a different scale, quantitative differences in SAA have been observed previously: The proportion of SAA-positive patient samples was found to differ between populations defined by genetic causes or clinical features such as normosmia [16, 40]. These findings already suggested that there is heterogeneity in SAA findings, even between patients with Lewy body disorders. Presently, we are not able to resolve whether the faster aggregation kinetics observed in samples from patients with lower cognitive performance reflects a more advanced disease stage or a more aggressive disease subtype. Moreover, we cannot resolve whether faster aggregation kinetics reflect a higher load of aSyn aggregates or strains with different biophysical properties. The lack of correlation of kinetic features with disease duration suggests the latter and indicates that SAA features could remain stable over time. Previously, we found no statistically significant change in the number of positive replicates over time [16], but an effect could be missed due to the low variance (average N/4 > 3). Longitudinal studies will be required to resolve this question. If kinetic features of the SAA indeed reflect disease progression, they could potentially be used as biomarkers to detect target engagement for therapies directed against aSyn pathology. Alternatively, kinetic features could be used to select patients with a more aggressive course of Lewy pathology for such therapies.

## Conclusions

The qualitative findings of the academic SAA used in this study are highly reproducible across laboratories following the same protocol. This adds to the already existing evidence for SAA being a future diagnostic tool in Lewy body disorders. After the implementation of further quality assurance measurements, the aSyn SAA will be translated to clinical routine diagnostic, as it has been for Creutzfeldt-Jakob disease.

Our study suggests that SAA may not only report the presence of Lewy pathology in a living patient. Rather, quantitative assay features could be associated with phenotypical patient characteristics, in particular the cognitive performance. Therefore, aSyn SAA can potentially be used for patient stratification and determining the target engagement of aSyn targeting treatments.

### Supplementary Information

**Additional file 1**. **Supplemental Table S1**: Individual patient characteristics and SAA-results.

**Additional file 2**.** Supplemental Figure S1**: Results of the inter- and intra-batch variability analysis. **Supplemental Figure S2**: Correlation of the average lag phase with clinical features. **Supplemental Figure S3**: Correlation of UPDRS-III with MoCA. **Supplemental Table S2**: Qualitative findings at TUD and ISNB. **Supplemental Table S3**: Number of positive replicates (N/4) at TUD and ISNB.

**Additional file 3**. List of R-codes.

## Data Availability

Pseudonymized data are available from the authors on reasonable request.

## Abbreviations

95% CI: 95% Confidence interval
aSyn: Alpha-synuclein
AUC: Area under curve
CSF: Cerebrospinal fluid
DLB: Dementia with Lewy bodies
Imax: Maximal fluorescence intensity
ISNB: Istituto delle Scienze Neurologiche di Bologna
LAG: Lag phase
MMSE: Mini mental status examination
MoCA: Montreal cognitive assessment
N/4: Number of positive replicates (out of four)
PC: Principal component
PD: Parkinson’s disease
RFU: Relative fluorescence unit
ROC: Receiver operating characteristic
SAA: Seed amplification assay
TUD: Technische Universität Dresden
UPDRS-III: Unified Parkinson’s disease rating scale part III
WT: Wild type
