# Supplementary material for: Kinetic parameters of alpha-synuclein seed amplification assay correlate with cognitive impairment in patients with Lewy body disorders
Source: Acta Neuropathol Commun. 2023 Oct 9;11:162. doi: 10.1186/s40478-023-01653-3 (PMC10563218; doi:10.1186/s40478-023-01653-3)
Supplement: Supplementary file 1 — Additional file 1. Supplemental Table S1: Individual patient characteristics and SAA-results. [file 40478_2023_1653_MOESM1_ESM.pdf]

Kinetic parameters of alpha-synuclein seed amplification assay correlate with cognitive impairment in patients with Lewy body disorders

Stefan Bräuer[1,2], Marcello Rossi[3], Johann Sajapin[4], Thomas Henle[4], Thomas Gasser[5,6], Piero Parchi[3,7], Kathrin Brockmann[5,6], Björn H. Falkenburger[1,2]

1) Department of Neurology, University Hospital Carl Gustav Carus at TU Dresden, Dresden, Germany
2) German Center for Neurodegenerative Diseases (DZNE), Dresden, Germany
3) IRCCS Istituto delle Scienze Neurologiche di Bologna (ISNB), Bologna, Italy
4) Department of Food Chemistry, TU Dresden, Dresden, Germany
5) Hertie Institute for Clinical Brain Research, Department of Neurodegenerative Diseases, Eberhard Karls University Tübingen, Tübingen, Germany
6) German Center for Neurodegenerative Diseases (DZNE), Tübingen, Germany
7) Department of Biomedical and Neuromotor Sciences, University of Bologna, Italy

Additional File 1

Supplemental Table S1: Individual patient characteristics and SAA-results

Supplemental Table S1: Individual patient characteristics and SAA-results

Patient	Sex	Diagnosis	Age	AAO	Duration	LEDD	UPDRS-III	MoCA	N/4	SAA-result	AUC	Imax	LAG	TT1	TT2
1	f	DLB	75.07	73.00	2.07	150.00		12	4	positive	3241686.11	78.53	22.31	21.75	22.5
2	m	DLB	75.20	72.00	3.20	200.00		5	4	positive	2093131.46	65.30	28.50	24	24
3	f	DLB	81.01	78.00	3.01	150.00		16	2	positive	1468537.36	48.85	31.50	24.75	38.25
4	f	DLB	63.60	58.00	5.60	604.00			0	negative					
5	f	DLB	75.90	74.00	1.90	150.00		10	4	positive	3702499.64	81.63	22.50	18	18.75
6	f	DLB	57.87	56.00	1.87				0	negative					
7	m	DLB	74.19	72.00	2.19			8	4	positive	3384051.03	70.86	21.19	19.5	19.5
8	m	DLB	62.40	59.00	3.40	488.00		20	3	positive	2946359.62	67.91	24.50	21.75	21.75
9	m	DLB	70.42	65.00	5.42	150.00	19	18	4	positive	4138953.85	82.42	20.25	18	20.25
10	m	DLB	64.16	52.00	12.16	850.00		5	4	positive	1428698.65	36.07	30.94	25.5	29.25
11	m	DLB	69.03	66.00	3.03	675.00			0	negative					
12	f	DLB	78.21	75.00	3.21	300.00		7	4	positive	2904304.36	67.12	24.38	21.75	23.25
13	m	DLB	64.51	62.00	2.51	200.00	58	14	2	positive	4919124.33	93.45	17.63	17.25	18
14	m	DLB	78.92	70.00	8.92	354.00		8	4	positive	3540988.75	79.20	21.94	18.75	21.75
15	m	DLB	69.26	67.00	2.26	663.00	27	4	4	positive	2615717.91	58.38	24.19	22.5	24
16	m	PD	68.83				36	10	4	positive	4715038.19	94.26	20.81	15	18.75
17	m	PD	77.19					19	2	positive	4789146.66	100.00	21.38	21	21.75
18	m	DLB	58.20	56.00	2.20		12	19	2	positive	984969.89	29.37	35.63	33	38.25
19	f	DLB	71.56	70.00	1.56		21	15	4	positive	3349724.78	74.92	22.88	20.25	21.75
20	m	DLB	75.93	74.00	1.93	500.00	22	9	3	positive	1525736.03	40.75	30.25	28.5	31.5
21	m	PD	65.03	61.00	4.03	300.00	28	28	0	negative					
22	m	DLB	68.58	67.00	1.58			19	2	positive	2174399.60	67.43	27.38	25.5	29.25
23	f	PD	65.44	60.00	5.44	153.00	10	30	0	negative					
24	m	DLB	65.28	64.00	1.28			20	4	positive	2527921.97	67.36	26.06	24.75	24.75
25	m	DLB	66.32	62.00	4.32		45	20	4	positive	1993250.67	57.02	28.13	23.25	25.5
26	m	DLB	77.56	76.00	1.56	300.00		19	3	positive	2969684.41	70.92	24.50	18.75	23.25

27	m	DLB	71.42	69.00	2.42	200.00			0	negative				
28	m	DLB	66.14	63.00	3.14	300.00	26	9	2	positive	2203810.07	50.44	25.50	25.5	26.25
29	f	PD	63.86	57.00	6.86		17	29	0	negative				
30	m	DLB	70.65	65.00	5.65	600.00			3	positive	2305174.57	57.94	26.00	23.25	27
31	m	DLB	76.02	72.00	4.02	200.00		13	4	positive	4147467.74	92.76	22.31	18	18
32	m	DLB	68.67	66.00	2.67	250.00	14	13	3	positive	2825911.83	61.74	23.25	21.75	23.25
33	m	DLB	73.33	71.00	2.33		60	16	4	positive	2848077.10	62.46	23.63	21.75	24
34	m	DLB	70.19	68.00	2.19		35	14	0	negative					
35	m	DLB	79.90	77.00	2.90	500.00	24	24	4	positive	1265625.43	34.60	32.44	30	31.5
36	m	PD	78.12	76.00	2.12	440.00	20	23	2	positive	1814893.00	43.00	25.50	24	27
37	m	PD	78.72	74.00	4.72	610.00	19	27	3	positive	1064656.66	36.50	32.00	24	35.25
38	m	PD	62.97	60.00	3.00		31		2	positive	1882414.45	52.81	29.25	25.5	33
39	m	PD	49.84	48.00	2.00		17	27	2	positive	4106976.08	94.92	24.38	18.75	30
40	f	PD	51.98	50.00	2.00		15	28	2	positive	965376.08	33.10	36.38	35.25	37.5
41	m	PD	67.08	66.00	1.00		15	30	3	positive	1634290.99	43.07	30.75	24	33.75
42	m	DLB	82.09	76.00	6.00		34	18	4	positive	2702812.13	74.56	25.31	22.5	24
43	m	DLB	78.17	75.00	3.00		38	6	4	positive	4343503.10	93.04	18.94	16.5	17.25
44	f	PD	50.98	49.00	2.00		21	29	2	positive	3151976.24	72.12	22.88	21	24.75
45	m	PD	50.60	48.00	2.00		12	29	3	positive	2714665.34	67.63	26.00	23.25	24
46	m	PD	59.30	55.00	4.00		7	25	3	positive	1989857.33	52.99	29.50	22.5	30.75
47	f	PD	63.48	56.00	7.00		7	27	3	positive	2044815.30	57.84	30.50	21.75	31.5
48	m	PD	59.97	57.00	3.00		22	27	2	positive	5044868.88	100.00	21.75	18	25.5
49	f	PD	83.11	80.00	3.00		14	27	2	positive	3390971.45	77.36	22.50	21	24
50	m	DLB	75.79						0	negative					
51	f	DLB	74.23					9	4	positive	4079874.13	78.18	20.06	18	19.5
52	m	DLB	72.43					10	3	positive	2197478.93	54.73	28.75	23.25	26.25
53	m	DLB	74.36					21	3	positive	2487518.36	69.59	25.75	23.25	27
54	m	DLB	81.75					4	3	positive	4317525.67	97.54	21.00	15	22.5
55	m	DLB						16	2	positive	2159980.73	70.75	26.25	23.25	29.25

56	m	PD	36.06	33.00	3.00		18	28	2	positive	1426508.02	57.96	32.63	28.5	36.75
57	m	PD	63.87	49.00	14.00		36	15	3	positive	4795432.47	93.24	20.00	15.00	18.00
58	f	PD	78.95	76.00	2.95	230.00	13	29	0	negative					
59	m	PD	60.68	56.00	4.68	415.00	5	29	3	positive	1018960.16	46.50	31.75	30	30
60	m	DLB	76.43	71.00	5.43	300.00	28	11	4	positive	5346562.92	100.0	18.19	16.5	18.75
61	m	PD	69.88	64.00	5.88	550.00	32	24	2	positive	3416496.95	73.55	21.00	20.25	21.75
62	f	PD	66.38	64.00	2.38	640.00	34	28	0	negative					
63	m	DLB	68.07	65.00	3.07	300.00		19	3	positive	1721470.73	48.00	29.75	26.25	27.75
64	m	PD	71.34	69.00	2.34	600.00	33	26	3	positive	889901.12	34.90	33.25	30	32.25
65	m	DLB	77.21	72.00	5.21	150.00	35	12	4	positive	2262004.22	74.18	28.31	21	24.75
66	f	DLB	70.47					15	4	positive	3190842.75	66.98	22.69	18.75	21.75
67	m	DLB	67.49						0	negative					
68	f	DLB	79.82					15	2	positive	1654934.58	39.85	31.50	27	36
69	m	DLB	77.21					13	2	positive	2639181.75	63.83	24.00	24	24
70	m	PD	74.65				47	18	2	positive	1621143.63	41.69	29.25	28.5	30
71	m	DLB	69.91	66.00	3.91	563.00			3	positive	3423360.10	83.06	23.25	21	24
72	f	DLB	84.01	82.00	2.01	873.00			0	negative					
73	m	PD	70.95				29	20	3	positive	2798877.53	65.15	25.00	21	23.25
74	m	PD	64.15	62.00	2.00		17	26	4	positive	2122387.03	64.90	27.38	24	27
75	m	PD	56.06				17	28	2	positive	2540996.17	73.63	27.38	22.5	32.25

Abbreviations: f, female; m, male; PD, Parkinson’s disease; DLB, Dementia with Lewy bodies; AAO, age at onset; duration, disease duration; LEDD, levodopa daily dose; UPDRS-III, Unified Parkinson’s disease rating scale part III; MoCA, Montreal cognitive assessment; SAA, seed amplification assay; N/4, number of positive replicates (out of four); AUC, area under the curve; Imax, maximum fluorescence intensity; LAG, mean lag phase; TT1, fastest lag phase; TT2, second fastest lag phase
