# Supplementary material for: Kinetic parameters of alpha-synuclein seed amplification assay correlate with cognitive impairment in patients with Lewy body disorders
Source: Acta Neuropathol Commun. 2023 Oct 9;11:162. doi: 10.1186/s40478-023-01653-3 (PMC10563218; doi:10.1186/s40478-023-01653-3)
Supplement: Supplementary file 2 — Additional file 2. Supplemental Figure S1: Results of the inter- and intra-batch variability analysis. Supplemental Figure S2: Correlation of the average lag phase with clinical features. Supplemental Figure S3: Correlation of UPDRS-III with MoCA. Supplemental Table S2: Qualitative findings at TUD and ISNB. Supplemental Table S3: Number of positive replicates (N/4) at TUD and ISNB. [file 40478_2023_1653_MOESM2_ESM.pdf]

Kinetic parameters of alpha-synuclein seed amplification assay correlate with cognitive impairment in patients with Lewy body disorders

Stefan Bräuer[1,2], Marcello Rossi[3], Johann Sajapin[4], Thomas Henle[4], Thomas Gasser[5,6], Piero Parchi[3,7], Kathrin Brockmann[5,6], Björn H. Falkenburger[1,2]

1) Department of Neurology, University Hospital Carl Gustav Carus at TU Dresden, Dresden, Germany
2) German Center for Neurodegenerative Diseases (DZNE), Dresden, Germany
3) IRCCS Istituto delle Scienze Neurologiche di Bologna (ISNB), Bologna, Italy
4) Department of Food Chemistry, TU Dresden, Dresden, Germany
5) Hertie Institute for Clinical Brain Research, Department of Neurodegenerative Diseases, Eberhard Karls University Tübingen, Tübingen, Germany
6) German Center for Neurodegenerative Diseases (DZNE), Tübingen, Germany
7) Department of Biomedical and Neuromotor Sciences, University of Bologna, Italy

Additional File 2:

Supplemental Figure S1: Results of the inter- and intra-batch variability analysis.

Supplemental Figure S2: Correlation of the average lag phase with clinical features.

Supplemental Figure S3: Correlation of UPDRS-III with MoCA.

Supplemental Table S2: Qualitative findings at TUD and ISNB.

Supplemental Table S3: Number of positive replicates (N/4) at TUD and ISNB.

Supplemental Figure S1: Results of the inter- and intra-batch variability analysis

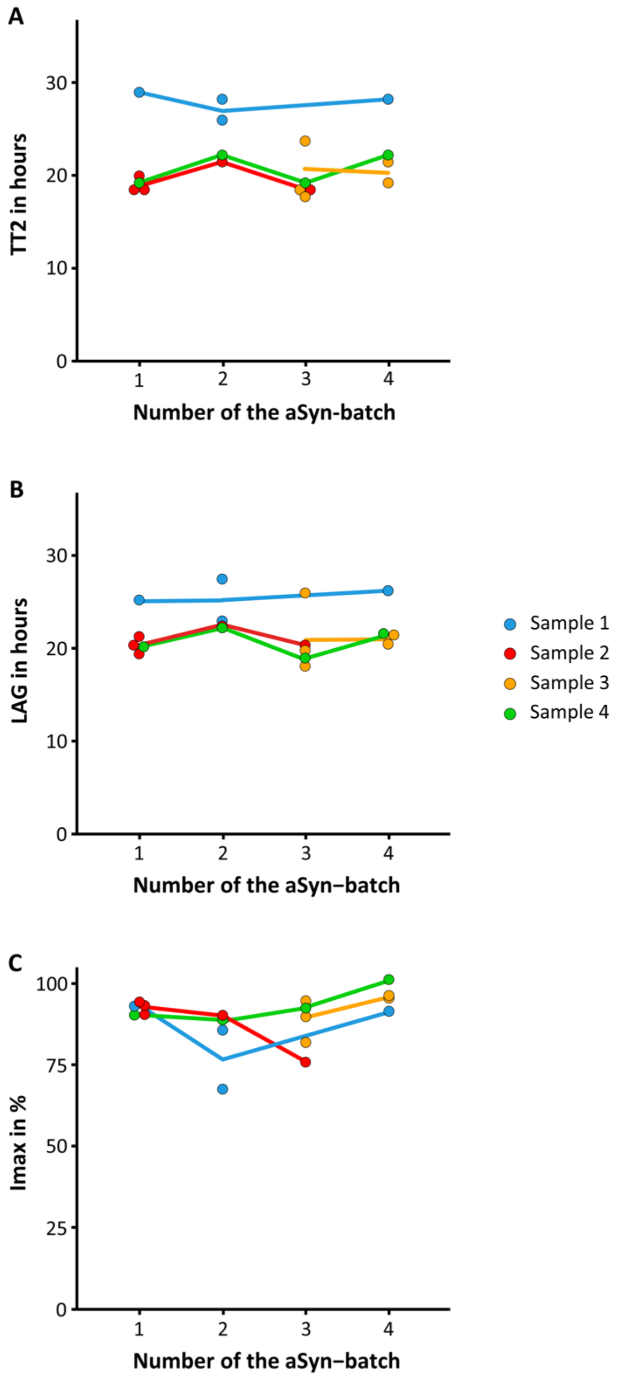

A Variation of the second fastest Lag phase (TT2) across different aSyn-batches. **B** Variation of the average lag phase (LAG) across different aSyn-batches. **C** Variation of the average maximum fluorescence intensity (Imax) across different aSyn-batches. In A-C, each marker represents the TT2/LAG/Imax of one measurement of a validation sample. We used four different samples in total each is depicted by one color (red, orange, green, blue). Lines connect the mean value of a sample in the respective batch.

Supplemental Figure S2: Correlation of the average lag phase with clinical features

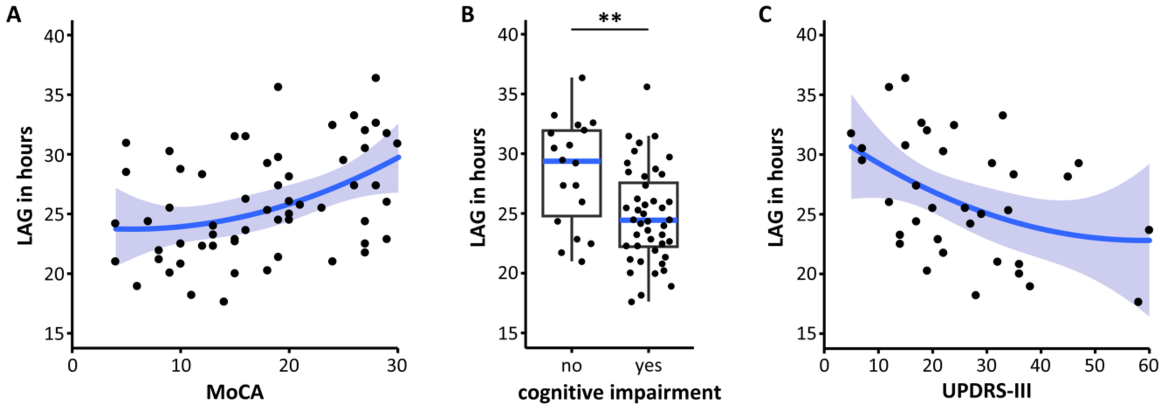

A Correlation of the average lag phase (LAG) with cognitive performance as quantified by the Montreal cognitive assessment (MoCA). **B** LAG for patients with and without cognitive impairment, defined as MoCA < 24. Comparison by unpaired t-test, d= 0.895, 95%CI= 0.324 – 1.465, p< 0.01. **C** Correlation of TT2 with motor symptoms reflected by the unified Parkinson's disease rating scale part III (UPDRS-III). In A-C, each marker represents one patient sample. In A and C, nonlinear regression and 95% confidential interval are indicated by blue line and blue-grey area. In B, box indicates 25 and 75 percentiles, whiskers indicate the range, blue line indicates median.

Supplemental Figure S3: Correlation of UPDRS-III with MoCA

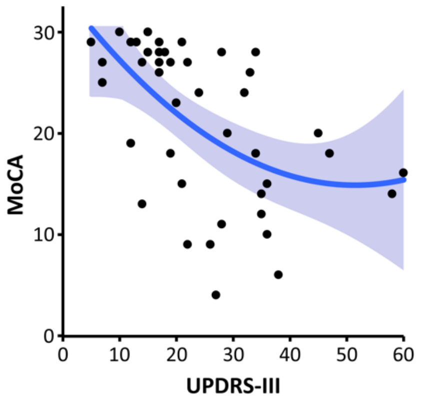

Correlation of motor symptoms reflected by the unified Parkinson's disease rating scale part III (UPDRS-III) with cognitive performance as quantified by the Montreal cognitive assessment (MoCA). Each marker represents one patient sample. Nonlinear regression and 95% confidential interval are indicated by blue line and blue-grey area.

Supplemental Table S2: Qualitative findings at TUD and ISNB

	Positive at TUD	Negative at TUD
Positive at ISNB	42	1
Negative at ISNB	0	12

Supplemental Table S3: Number of positive replicates (N/4) at TUD and ISNB

	0 of 4 at TUD	2 of 4 at TUD	3 of 4 at TUD	4 of 4 at TUD
0 of 4 at ISNB	12	0	0	0
2 of 4 at ISNB	0	0	1	0
3 of 4 at ISNB	1	4	5	2
4 of 4 at ISNB	0	6	7	17
