# Supplementary material for: Kinetic parameters of alpha-synuclein seed amplification assay correlate with cognitive impairment in patients with Lewy body disorders
Source: Acta Neuropathol Commun. 2023 Oct 9;11:162. doi: 10.1186/s40478-023-01653-3 (PMC10563218; doi:10.1186/s40478-023-01653-3)
Supplement: Supplementary file 3 — Additional file 3. List of R-codes. [file 40478_2023_1653_MOESM3_ESM.pdf]

Kinetic parameters of alpha-synuclein seed amplification assay correlate with cognitive impairment in patients with Lewy body disorders

Stefan Bräuer[1,2], Marcello Rossi[3], Johann Sajapin[4], Thomas Henle[4], Thomas Gasser[5,6], Piero Parchi[3,7], Kathrin Brockmann[5,6], Björn H. Falkenburger[1,2]

1) Department of Neurology, University Hospital Carl Gustav Carus at TU Dresden, Dresden, Germany
2) German Center for Neurodegenerative Diseases (DZNE), Dresden, Germany
3) IRCCS Istituto delle Scienze Neurologiche di Bologna (ISNB), Bologna, Italy
4) Department of Food Chemistry, TU Dresden, Dresden, Germany
5) Hertie Institute for Clinical Brain Research, Department of Neurodegenerative Diseases, Eberhard Karls University Tübingen, Tübingen, Germany
6) German Center for Neurodegenerative Diseases (DZNE), Tübingen, Germany
7) Department of Biomedical and Neuromotor Sciences, University of Bologna, Italy

Supplemental Text:

R code for figures and analyses

<u>##Spearman correlation</u>

```
library(readxl)

dat<-read_xlsx("C:/… XXX.xlsx")

cor.test(dat$k_Moca,dat$DD_tt2,method="spearman")

cor.test(dat$k_Moca,dat$DD_LAG,method="spearman")

cor.test(dat$k_UPDRS3,dat$DD_tt2,method="spearman")

cor.test(dat$k_Moca,dat$k_UPDRS3,method="spearman")
```

<u>## test for normal distribution and Mann-Whitney U test</u>

```
library(readxl)

data <-read_xlsx("C:/… XXX.xlsx")

data$dementia <- as.factor(data$dementia)

head(data)

shapiro.test(data$TT2)
```

```
with(data, shapiro.test(data$TT2[dementia == "0"]))

with(data, shapiro.test(data$TT2[dementia == "1"]))

wilcox.test(TT2~dementia, data = data, exact = FALSE, correct = FALSE, conf.int = TRUE)

z <- qnorm(p/2)

r <- z/sqrt(n)

z

r
```

<u>## test for normal distribution and unpaired t-test</u>

```
library(readxl)

data <-read_xlsx("C:/… XXX.xlsx")

data$dementia <- as.factor(data$dementia)

head(data)

shapiro.test(data$LAG)

with(data, shapiro.test(data$LAG[dementia == "0"]))

with(data, shapiro.test(data$LAG[dementia == "1"]))

res <- t.test(LAG~dementia, data = data, var.equal = TRUE)

res
```

<u>## boxplot figure 2B</u>

```
library(ggplot2)

library(readxl)

data <-read_xlsx("C:/… XXX.xlsx")

p <- ggplot(data, aes(x=dementia, y=TT2)) +

  geom_boxplot(outlier.shape = NA) +

  stat_summary(fun.y=mean, geom="errorbar", width = .75, linetype = "dashed", color="red",
aes(ymax =..y.., ymin =..y..)) +

  geom_jitter(alpha=0.8, width=0.3, size=2, colour="black") +
```

```
  ylim(15, 40) +
  theme_classic()
p
```

boxplot supplemental figure S2B

```
library(ggplot2)
data <-read_xlsx("C:/… XXX.xlsx")
p <- ggplot(data, aes(x=dementia, y=LAG)) +
  geom_boxplot(outlier.shape = NA) +
  stat_summary(fun.y=mean, geom="errorbar", width = .75, linetype = "dashed", color="red", 
aes(ymax =..y.., ymin =..y..)) +
  geom_jitter(alpha=0.8, width=0.3, size=2, colour="black") +
  ylim(15, 40) +
  theme_classic()
p
```

figure 2A

```
library(ggplot2)
library(readxl)
test2 <-read_xlsx("C:/… XXX.xlsx")
ggp <- ggplot(test2, aes(Moca,TT2)) +
  geom_point() +
  xlim(0,30) + ylim(15,40) +
  scale_x_continuous(expand = c(0, 0), limits=c(0, NA))
ggp
ggp+ stat_smooth(method= "lm",formula= y ~ poly(x,2),) +
theme_classic()
```

supplemental figure S2A

```
library(ggplot2)
library(readxl)
test2 <-read_xlsx("C:/… XXX.xlsx")
ggp <- ggplot(test2, aes(Moca,LAG)) +
  geom_point() +
  xlim(0,30) + ylim(15,40) +
  scale_x_continuous(expand = c(0, 0), limits=c(0, NA))
ggp
ggp+ stat_smooth(method= "lm",formula= y ~ poly(x,2),) +
theme_classic()
```

##figure 2C and supplemental figure S2C

```
library(ggplot2)
library(readxl)
test2 <-read_xlsx("C:/… XXX.xlsx")
tt2<-test2$DD_tt2
lag<-test2$DD_LAG
updrs<-test2$k_UPDRS3
ggp <- ggplot(test2, aes(updrs,tt2)) +
  geom_point() +
  xlim(0,65) + ylim(14,40) +
  scale_x_continuous(expand = c(0, 0), limits=c(0, NA))
ggp
ggp+ stat_smooth(method= "lm",formula= y ~ poly(x,2),) +
  theme_classic()
ggp <- ggplot(test2, aes(updrs,lag)) +
  geom_point()+
```

```
  xlim(0,65) + ylim(15,40) +
  scale_x_continuous(expand = c(0, 0), limits=c(0, NA))
ggp
ggp+ stat_smooth(method= "lm",formula= y ~ poly(x,2),) +
  theme_classic()
```

##supplemental figure S3

```
test2 <-read_xlsx("C:/… XXX.xlsx")
moca<-test2$Moca
updrs<-test2$UPDRS3
ggp <- ggplot(test2, aes(updrs,moca)) +
  geom_point()+
  xlim(0,65) + ylim(0,31) +
  scale_x_continuous(expand = c(0, 0), limits=c(0, NA))
ggp
ggp+ stat_smooth(method= "lm",formula= y ~ poly(x,2),) +
  theme_classic()
```

linear model

```
ana<-read.table(file = "XXX.csv", header = TRUE, na.strings = "NA", sep = ";", dec = ".")
f1 <- lm(DD_tt2 ~ 1, data=ana)
f2 <- lm(DD_tt2 ~ k_Moca, data=ana)
f3 <- lm(DD_tt2 ~ k_Moca + k_UPDRS3, data=ana)
anova(f1, f2, f3)
f1 <- lm(DD_tt2 ~ 1, data=ana)
f2 <- lm(DD_tt2 ~ k_UPDRS3, data=ana)
f3 <- lm(DD_tt2 ~ k_Moca + k_UPDRS3, data=ana)
anova(f1, f2, f3)
```

principal component analysis figure 1C

```
library(tidyr)
library(dplyr)
library(ggplot2)
library(hablar)
library(stringr)
library("FactoMineR")
library("factoextra")
library("corrplot")
setwd("C:/…")
d<-read.table(file = "XXX.csv", header = TRUE, na.strings = "NA", sep = ";", dec = ".")
summary(d)
head(d)
ana <- d
res.pca <- PCA(ana, graph = TRUE, scale.unit = TRUE, ncp = 9)
eig.val <- get_eigenvalue(res.pca)
eig.val
var <- get_pca_var(res.pca)
corrplot(var$cos2, is.corr=FALSE, cl.ratio = 1, cl.align = "r", cl.lim = c(0, 1),)
```

power analysis

```
library(pwr)
pwr.r.test(n = NULL, r = 0.4, sig.level = 0.05, power = 0.9, alternative = c("two.sided"))
```

cohen´s kappa

```
library(readxl)
dat<-read_xlsx("C:/… XXX.xlsx")
```

```
library(DescTools)

resulttab <- xtabs (~ dat$TUD + dat$ISNB)

resulttab

CohenKappa(resulttab, conf.level= 0.95)
```
